# USING PROCESS EVALUATION TO IMPROVE DOCUMENTATION OF LIFE-SUSTAINING TREATMENTS FOR VETERANS

**DOI:** 10.1093/geroni/igad104.2301

**Published:** 2023-12-21

**Authors:** Leah Haverhals, Kate Magid, Jennifer Kononowech, Jazmin Beltran, Amber Lane, Cari Levy

**Affiliations:** Department of Veterans Affairs Rocky Mountain Regional VA Medical Center, Denver, Colorado, United States; Veterans Health Administration, Aurora, Colorado, United States; VA Ann Arbor, Ann Arbor, Michigan, United States; Department of Veterans Affairs, Aurora, Colorado, United States; Rocky Mountain Regional VA Medical Center, Aurora, Colorado, United States; University of Colorado, Denver, Colorado, United States

## Abstract

The Preferences Elicited and Respected for Seriously Ill Veterans through Enhanced Decision-Making (PERSIVED) intervention began in 2021 to support United States Department of Veterans Affairs (VA) Home Based Primary Care (HBPC) clinicians in completing life-sustaining treatment (LST) templates for Veterans. In PERSIVED, HBPC clinicians partner with the PERSIVED team for 15 months, with clinicians routinely receiving coaching from the PERSIVED team. To assess participation in PERSIVED and to inform coaching sessions, the PERSIVED team conducted process evaluation interviews from August-November 2022 with N=24 clinicians across the country from seven HBPC sites. Interviews were conducted between months six-seven of PERSIVED implementation. Two PERSIVED team members conducted interviews, one acting as interviewer and the other as notetaker. Following interviews, the notetaker cleaned up the notes and the interviewer reviewed them, with the two reaching consensus on summaries on individual interviews and ultimately summarizing interview data by site. Two PERSIVED coaches then reviewed the summaries to brainstorm how to adapt coaching based on process evaluation findings. Coaches noted site-specific barriers and facilitators to improving LST template completion rates; positives of PERSIVED coaching; suggestions to improve PERSIVED; and goals for the remaining nine months of the PERSIVED intervention. Strategies suggested based on the process evaluation analyses included how to integrate checking for the LST template into existing processes, adapting note templates, discussing LST templates during team meetings, and improving education around LST template completion. The rapid integration of process evaluation findings into PERSIVED coaching sessions proved beneficial for participating HBPC teams.

